# Modeling and optimization of degree of folate grafted on chitosan and carboxymethyl-chitosan

**DOI:** 10.1007/s40204-015-0044-0

**Published:** 2015-11-19

**Authors:** S. Esfandiarpour-Boroujeni, S. Bagheri-Khoulenjani, H. Mirzadeh

**Affiliations:** 1 Department of Polymer and Color Engineering, Amirkabir University of Technology, 424, Hafez St., P.O. Box: 15875-4413, Tehran, Iran; 2 Cancer Model Research Centre, Cancer Institute of Iran, Tehran University of Medical Sciences, Tehran, Iran

**Keywords:** Folic acid modified chitosan, Carboxymethyl chitosan, Modeling of substitution degree, Design experiments

## Abstract

Chitosan is a cationic polysaccharide with great properties and so is considered as an attractive biopolymer. However, chitosan shows its antibacterial activity only in acidic environment and this restricts its uses. So water-soluble chitosan derivatives such as carboxymethyl chitosan could be good candidates for such biomedical applications. Modified chitosan with hydrophobic functional groups such as folate (FA) is able to make self-assembled nanoparticles in aqueous media. One of the most important factors affecting the properties of resulting nanoparticles such as size, morphology, amount and efficiency of drug loading and also drug release profile is the amount of FA groups grafted on the chitosan chains. In this study FA modified chitosan and carboxymethyl chitosan have been synthesized using folic acid, *N*-hydroxy succinimide (NHS), *N*, *N*-dicyclohexylcarbodiimide (DCC) and 1-ethyl-3-(3-dimethylaminopropyl) carbodiimide (EDC). The effect of molecular weight, degree of substitution of carboxymethyl hydrophilic group and primary molar ratio of folic acid to chitosan/carboxymethyl chitosan (CMCS) on degree of substitution of folate functional groups grafted on chitosan chains was modeled using a statistical software package (Design of Expert 8, Trial version). Degree of substitution of grafted folate was measured using UV/Vis spectroscopy. Results show that degree of substitution of CMC and molar ratio of folic acid to chitosan/carboxymethyl chitosan has direct effect on substitution degree of folate and molecular weight has an inverse impact. Also results show that molar ratio of folic acid to chitosan/(CMCS) has the most effect on substitution degree of folate and the proposed model is statistically valid to predict degree of substitution of FA groups on chitosan chains.

## Introduction

Chitosan (deacetylated chitin) is a biodegradable cationic polysaccharide consisting of random (1,4) of *N*-acetyl-d-glucosamine and d-glucosamine groups (Novoa-Carballal et al. 2013). Because of low toxicity, biodegradability, biocompatibility and high mucoadhesion, chitosan is considered as an attractive biopolymer for drug delivery systems (Prabaharan 2015) and tissue engineering applications (Jaikumar et al. 2015). Recently, anti-bacterial and anti-fungal activity of chitosan is widely considered. Chitosan prevents from the growth of several types of bacteria and fungus and shows a wide range of antibacterial activity, high speed killing and low toxicity for mammalian cells (de Oliveira et al. 2014). However, chitosan (p*K*a = 6.8) shows its antibacterial activity only in acidic environment that is due to its low solubility in the pH above 6.5. Water-soluble chitosan derivatives such as carboxymethyl chitosan that have solubility in the wide scale of pH in the range of 3–11 could be good candidates for such applications (Ge and Luo 2005; Nam et al. 2013).

In recent years, self-assembled polysaccharide hydrogel nanoparticles, especially chitosan nanoparticles, have been of interest due to their accumulate properties. Chemical modifying of chitosan with hydrophilic groups such as carboxymethyl is an important feature to form self-assembled nanoparticles (Tan and Liu 2011). Hydrophilic modification affects the formation and stability of the chitosan electrostatic complex. In addition, it is common that the conformational flexibility facilitates the formation of self-assembled nanostructures in solution, while molecular stiffness favors spatially ordered chitosan nanostructures (Yang et al. 2014).

Chitosan modified with hydrophobic molecules is capable to form hydrogel nanoparticles through intra-molecular and/or intermolecular interactions between hydrophobic sectors in an aqueous environment (Majedi et al. 2013). Active targeting has been attempted to gain a high degree of selectivity to a specific organ and to enhance the internalization of drug-loaded nanoparticles into the target cells. The internalization of drug-loaded nanoparticles is beneficial for more efficient drug therapy since the drug can be delivered directly to the target cells. Some hydrophobic groups such as folate will not only assist the stability of the nanoparticles, but also give them some special features such as targeting (Zhao et al. 2008).

Folic acid is a vitamin with very low solubility in water (0.0016 mg/ml in 25 °C) and shows high affinity to its receptor which is over-expressed in the cancerous cells. Thus, introduction of folate groups to the chitosan chains not only assists the polymeric chains to self-assemble but also gives the nanoparticles ability for targeting therapy (Zhao et al. 2008).

Degree of substitution of hydrophobic groups affect the final properties of nanoparticles including morphology, size, content and efficiency of drug loading and drug release profile (Novoa-Carballal et al. 2013).

In this paper, the effect of varying molecular weight, degree of substitution of hydrophilic groups and the molar ratio of folic acid to chitosan/carboxymethyl chitosan (CMCS) on the degree of substitution of folate groups grafted on chitosan and CMCS chains) are investigated using design experiment (two-level factorial model). The purpose of this paper was to model the percent of FA grafted on chitosan and CMCS. It should be noted that no report has been done on optimization and modeling of the degree of substitution of FA on chitosan.

## Experimental

### Materials

Chitosan with low molecular weight and DD = 89 % from Fluka, hydrogen peroxide 30 %, isopropyl alcohol, sodium chloride, sodium hydroxide, acetic acid, hydrochloric acid, dimethyl sulfoxide (DMSO), ethanol, sodium acetate, Monochloroacetic acid, *N*-hydroxy Succinimide (NHS) and *N*, *N*-dicyclohexylcarbodiimide (DCC) from Merck, folic acid (FA) from Roth and 1-ethyl-3-(3-dimethylaminopropyl) carbodiimide (EDC) from the Alfa Aesar company were perchased and used.

### Methods

#### Design experiment

In this research, design of experiment was performed using two-level factorial method with three variables (molecular weight, percent of carboxymethyl substitution and molar ratio of folic acid to NH_2_ groups of chitosan and CMCS) in two levels (molecular weight: 40 and 400 kD, percent of carboxymethyl substitution: 0 and 60 %, and molar ratio of folic acid to NH_2_ groups of chitosan and CMCS: 1 or 16). Table 1 shows the designed experiments. The least squares method was applied to generate models for predicting the degree of FA substitution. Analysis of variance (ANOVA), using a statistical software package (Design of Expert 8, Trial version), was applied to investigate the validity of the generated models based on experimental results and determine the main effects and interactions of factors on the response of model (Bagheri-Khoulenjani et al. 2009).

**Table 1:** The designed experiments, observed and estimated degree of FA substitution for each experiment by model

Sample	Molecular weight (KD)	Percentage of CMCS substitution	Molar ratio of folic acid to chitosan or carboxymethyl chitosan	Percentage of FA substitution	
				Observed value	Estimated value
1	400	0	16	51.43 ± 0.15	51.34
2	40	0	16	51.76 ± 0.21	51.85
3	40	60	16	52.82 ± 0.18	52.73
4	400	60	1	12.67 ± 0.14	12.93
5	40	0	1	12.02 ± 0.17	12.28
6	40	60	1	17.25 ± 0.14	16.99
7	400	60	16	52.12 ± 0.19	52.21
8	400	0	1	8.49 ± 0.23	8.23

### Synthesis of chitosan-FA

#### Degradation of chitosan

To reduce the molecular weight of chitosan from 400 KD to 40 KD, degradation of chitosan was performed using hydrogen peroxide. First 2 % (w/v) chitosan solution was prepared in1.2 M HCl. Then 4.4 ml hydrogen peroxide was added to the resulting solution and the reaction was carried out for 1.5 h at 30 °C. After the reaction, pH was raised to 7 with the addition of 5 M NaOH. The resulting precipitate was isolated by vacuum filter and washed with ethanol. The precipitate was dried overnight at room temperature.

#### Preparation of carboxymethyl chitosan (CMCS)

In order to prepare carboxymethyl chitosan, first 1 g chitosan was dissolved in 40 ml distilled water and 0.5 ml acetic acid. 1 M NaOH solution was added to chitosan solution while stirring promptly to precipitate chitosan. The precipitate was filtered and washed with distilled water. After filtering chitosan, it was distributed in 20 ml isopropanol, and 2 g NaOH powder was added and the mixture homogenized for 4–5 h with a mechanical stirrer. 2 g chloroacetic acid was dissolved in 5 ml isopropyl alcohol and was added to chitosan. The resulting solution stirred for 8 h at room temperature. The resulting precipitate was isolated and washed at least three times with ethanol/water at a ratio of 1:3, and dried for 24 h at ambient temperature.

#### Preparation of chitosan and carboxymethyl chitosan modified by folic acid

In order to prepare modified polymers with folate groups, folic acid was dissolved in dimethyl sulfoxide (DMSO) and activated by 0.1 g of NHS and 0.2 g of DCC for 12 h until folic acid was well dissolved (FA/NHS/DCC molar ratio = 1:2:2). It was then added to a solution of 1 % (w/v) CMCS (10 ml) and chitosan (7 ml) in buffer (pH 4.7). EDC (FA/EDC molar ratio = 1:1) was added to the resulting mixture and stirred for 16 h. pH was brought to 9.0 by dropwise addition of diluted aqueous NaOH and dialyzed first against phosphate buffer pH 7.4 for 1 day and then against water for 1 day. The resulting polymer was dried overnight at room temperature.

### Characterization methods

#### Viscometry

Molecular weight of chitosan and degraded chitosan was measured by Ubbelohde viscometer using 0.1 M CH3COOH/0.2 M NaCl solvent system at 25° C. Molecular weight was calculated by Mark–Houwink equation using *a* = 0.93 and *k* = 0.00181 ml/g (Bagheri-Khoulenjani et al. 2009).

#### Chemical structure of modified chitosan

FT-IR analysis (Bruker FRA model 106/5) was applied to study chemical composition of chitosan and modified chitosan samples. The infrared spectrum was obtained by KBr tablet method in wavenumber range of 4000–400 cm^−1^.

#### The degree of substitution (DS) of carboxymethyl group

The DS value of each sample was measured by potentiometric titration using pH meter (Metrohm, Switzerland). Dry CMC (0.20 g) was dissolved in 20.00 ml of hydrochloric acid standard solution (0.1000 mol/l). A standard solution of sodium hydroxide was used for titration. The following equation was used to calculate the total DS of the sample (Sun and Wang 2006):

1 $$ {\text{Total}}\;{\text{DS}} = \frac{{\left( {V_{2} - V_{1} } \right) \times C \times {\text{molar }}\;{\text{weight }}\;{\text{of }}\;{\text{chitosan }}\;{\text{residue}}}}{{1000\;{\text{W}}}}, $$

where *C* is the concentration of sodium hydroxide standard solution (mol/l), *V*_1_ the sodium hydroxide volume for titrating excessive hydrochloric acid (ml), and *V*_2_ is the volume corresponding to titration terminal of COOH and *W* is the weight of the sample (g).

#### The degree of substitution (DS) of folate group

The degree of substitution of FA into amino groups in final hydrophobic modified chitosan was determined by a UV/vis spectrophotometer (Analytik Jena, Germany) at 363 nm. Briefly, accurately weighed folate derivative was dissolved in water and DMSO(1/1:v/v) to obtain a 0.02 wt% CMC–FA conjugate solution, and then its optical density was measured with UV/vis spectrophotometer. FA powder was dissolved and diluted to a series of gradient FA standard solutions that were used to prepare the calibration curve. The degree of substitution (DS) was calculated according to the following formula (Wang et al. 2011):

2 $$ {\text{DS}} = \frac{{C/M_{\text{FA}} }}{{(m - c)/M_{\text{CMCS \,or\, chitosan}} }}, $$

where *c* is the content of the FA determined from the corresponding calibration curve; m is the amount of the modified polymers used in experiment; *M*_FA_ is the molecular weight of the FA and *M*_CMCS_ is the molecular weight of CMC and chitosan units.

## Results and discussion

### Chitosan degradation

**Fig. 1:**
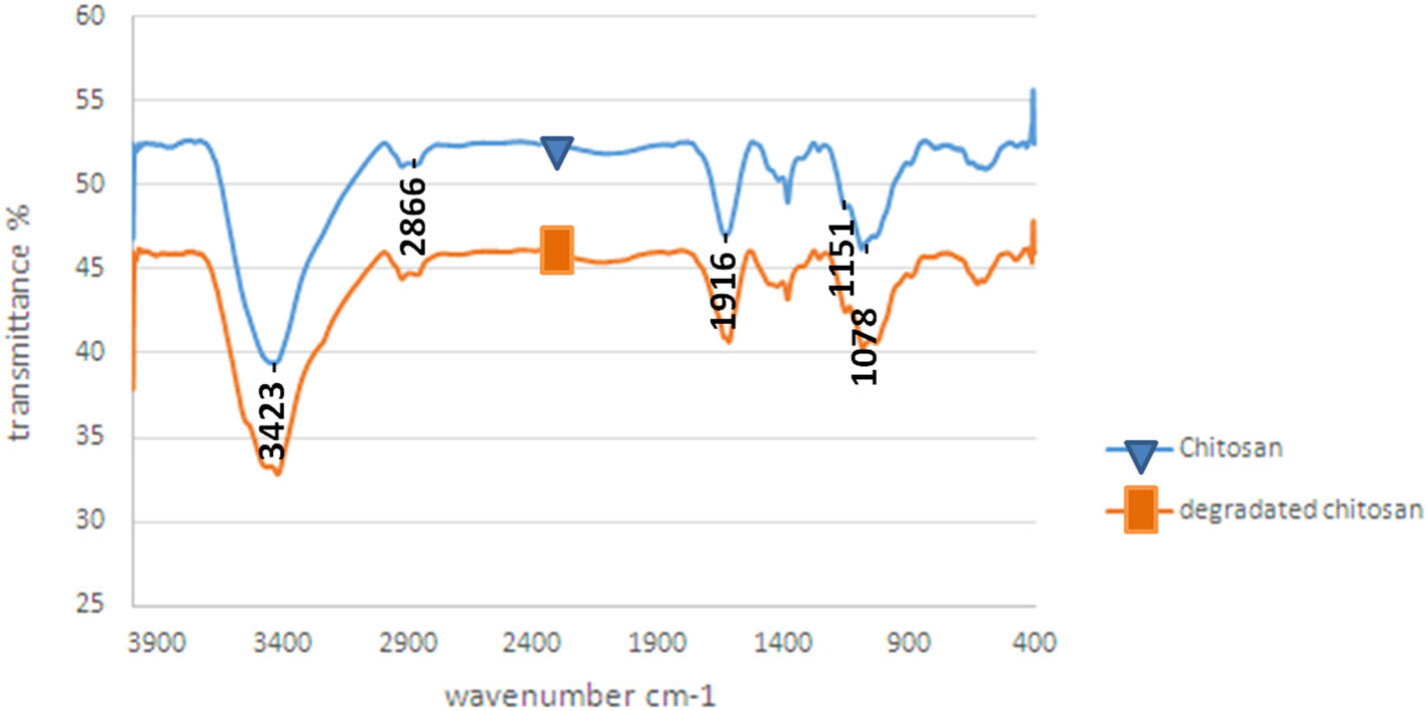
FTIR images of chitosan and degraded chitosan

The results of infrared spectroscopy for chitosan and degraded chitosan, as shown in Fig. 1, depict that in the process of degradation there were no distinguished changes in the Chitosan structure during the degradation process. In addition, the results of viscometry (Table 2) show that molecular weight of chitosan reduced to about one-tenth of its original value.

**Table 2:** The molecular weight of chitosan and degraded chitosan obtained by Ubbelohde viscometer

Chitosan molecular weight (KD)	Degraded chitosan molecular weight (KD)
433,200 ± 22,000	43,500 ± 2800

### Chemical structure of modified chitosan

**Fig. 2:**
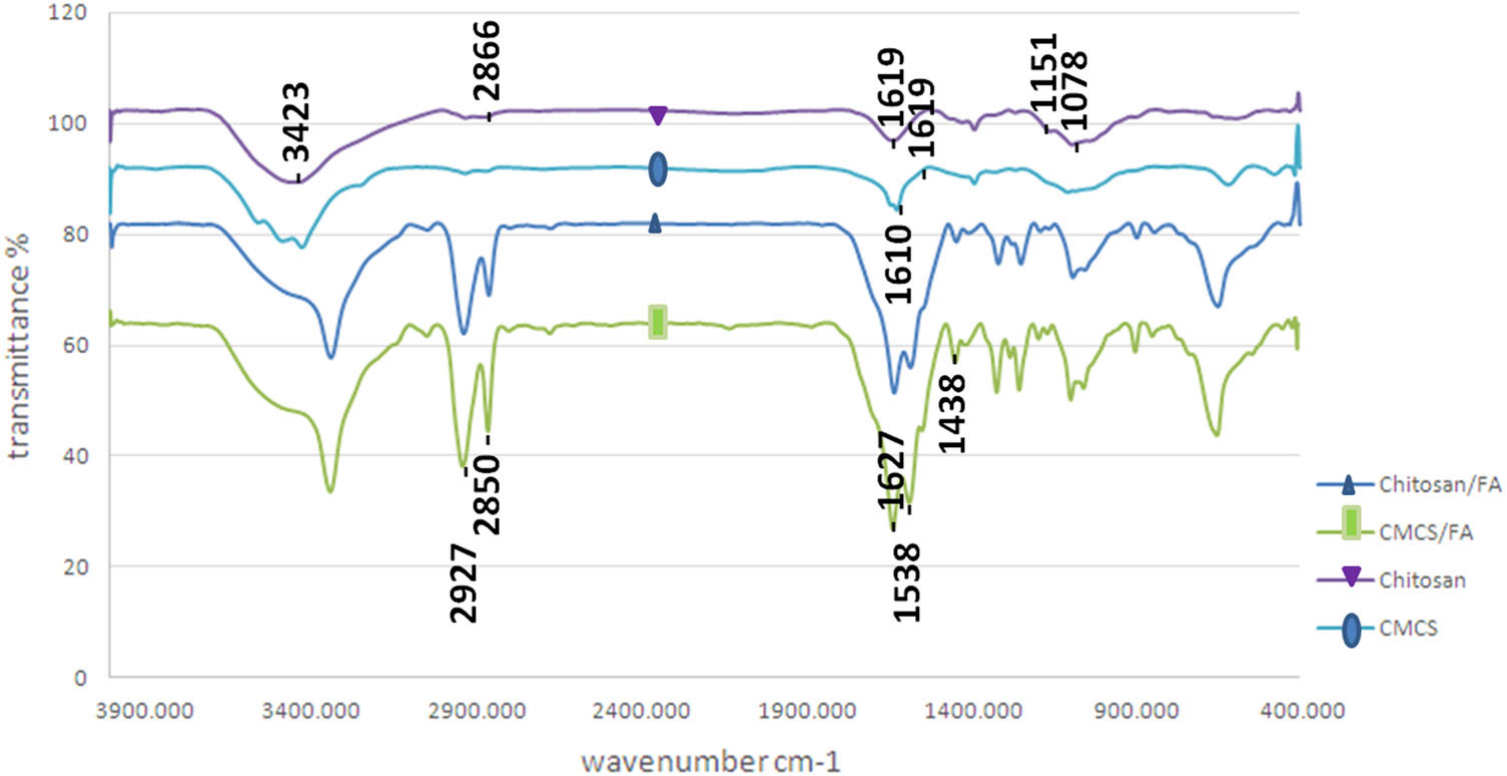
FTIR spectra of chitosan, carboxymethyl chitosan, chitosan with folate group, carboxymethyl chitosan with folate group

**Fig. 3:**
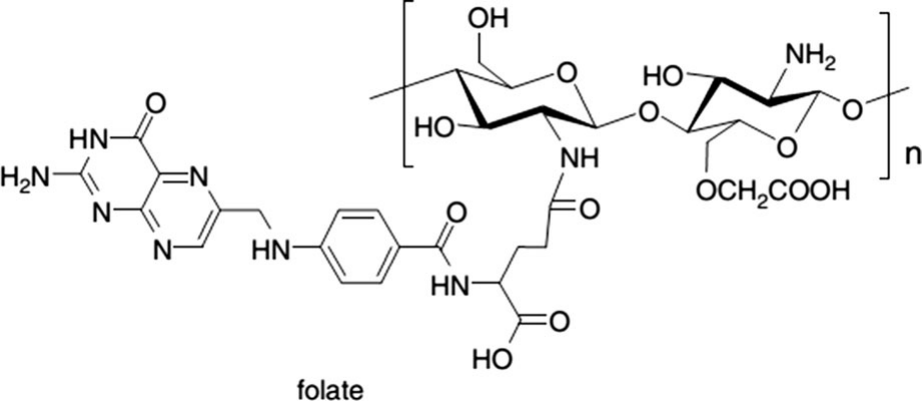
A scheme of chemical structure of the synthesized carboxymethyl chitosan with folate group

FTIR spectrum of chitosan (Fig. 2) shows all the characteristic peaks of chitosan including 3423 cm^−1^ (OH stretch), 2866 cm^−1^ (CH stretch), 1619 cm^−1^ (NH bending), 1151 cm^−1^ (stretch of O bridges) and 1078 cm^−1^ (CO stretch). Comparison of the spectra of CMCS and chitosan revealed a new peak in 1610 cm^−1^ (related to C=O) which confirms introduction of carboxymethyl functional groups into the chitosan chains. FTIR spectrum of chitosan and carboxymethyl chitosan modified with folic acid show various peaks corresponding to folate group at 1438, 1538, 1627, 2850 and 2927 cm^−1^. Absorption peaks in 1438 and 1627 cm^−1^ were related to the stretching vibration of C=C in aromatic ring. Carbonyl absorption peak, C=O, known as amide I peak and N–H called amide II. Amide II bond observed in relatively high frequencies (1577 cm^−1^) that occurs due to the formation of hydrogen bonds (Yang et al. 2010; Sahu et al. 2010). A scheme of chemical structure of the synthesized carboxymethyl chitosan with folate group is shown in Fig. 3.

### Modeling of FA degree of substitution

To model the degree of substitution of FA groups onto the chitosan chains within the design space, the first-order model (Montgomery and Montgomery 1984) has been used as following:

3 $$ y = b_{0} + b_{1} x_{1} + b_{2} x_{2} + {\text{b}}_{3} {\text{x}}_{3} + b_{1,3} x_{1} x_{3} + b_{2,3} x_{2} x_{3 } + b_{1,2} x_{1} x_{2} , $$

where *b*_0_ is the arithmetic mean response of 9 runs and *b*_1_, *b*_2_ and *b*_3_ are the estimated coefficients for the factors *x*_1_, *x*_2_ and *x*_3_, respectively. The main effects represent the average results of changing one factor at a time from its lowest to highest values. The interactions show the changes in particle size where two or more factors vary simultaneously (Bhavsar et al. 2006).

The proposed model for estimating the degree of FA substitution (*y*) in terms of molecular weight (*x*_1_), percentage of CMC substitution (*x*_2_) and molar ratio of folic acid to chitosan and carboxymethyl chitosan (*x*_3_) based on experimental data and after removal of non-effective terms is developed as shown in Eq. (4):

4 $$ y = 10.12128 - 0.011919x_{1} + 0.082672x_{2} + 2.61178x_{3} + 6.55556 \times 10^{ - 4} x_{1} x_{3} - 4.25556 \times 10^{ - 3} x_{2} x_{3} . $$

To investigate validity of the proposed model, the *P* value, *R* ^2^, predicted and adjusted-*R* ^2^ and precision adequacy value were calculated (Table 3). *P* value of proposed model is equal to the 0.0002 and less than 0.05 that represents the model is significant. *R* ^2^, predicted and adjusted *R* ^2^values are 0.9999, 0.9997 and 0.9984, respectively. *R* ^2^ values are all close to 1 and predicted and adjusted *R* ^2^ values are compatible. To study the validity of model, the precision adequacy can be used. Its values more that 4 confirm the validity of the model. The adequacy of the proposed model is 130.548 that represents model is valid and desirable (Bhavsar et al. 2006). In addition, in Table 4, *P* values of the proposed model terms has been presented and all of them are under 0.05.

**Table 3:** ANOVA of proposed model to estimate degree of FA substitution

	*P* vlaue of model	*R* ^2^	*R* ^2^		Precision adequacy
			Adjusted	Predicted	
Modified first-order model	0.0002	0.9999	0.9997	0.9984	130.548

**Table 4:** P value of different terms in the proposed model

Term	*x*_1_	*x*_2_	*x*_3_	*x*_1_ *x*_3_	*x*_1_ *x*_2_
*P* value	0.0145	0.0098	<0.0001	0.0238	0.0235

To test the model out of its original points, some samples were synthesized and their degree of FA substitution was compared to the ones predicted by the Eq. (4) (Table 5). As the results show, there is a good agreement between experimental data and the predicted ones. All these data show the validity of the proposed model.

**Table 5:** Experimental and estimated degree of FA substitution by model for samples out of the design points

Sample	Molecular weight (KD)	Percentage of CMCS substitution	Molar ratio of folic acid to chitosan or carboxymethyl chitosan	Percentage of FA substitution	
				Observed value	Estimated value
O1	400	0	2	12.37 ± 0.18	11.10
O2	400	0	10	31.07 ± 0.28	34.09
O3	40	0	4	19.11 ± 0.29	20.20
O4	40	0	6	31.74 ± 0.24	25.47
O5	400	60	5.5	19.56 ± 0.11	24.72
O6	40	60	0.8	12.54 ± 0.25	16.51
O7	40	60	4.8	22.61 ± 0.13	26.04
O8	40	60	14	34.82 ± 0.12	47.96

**Fig. 4:**
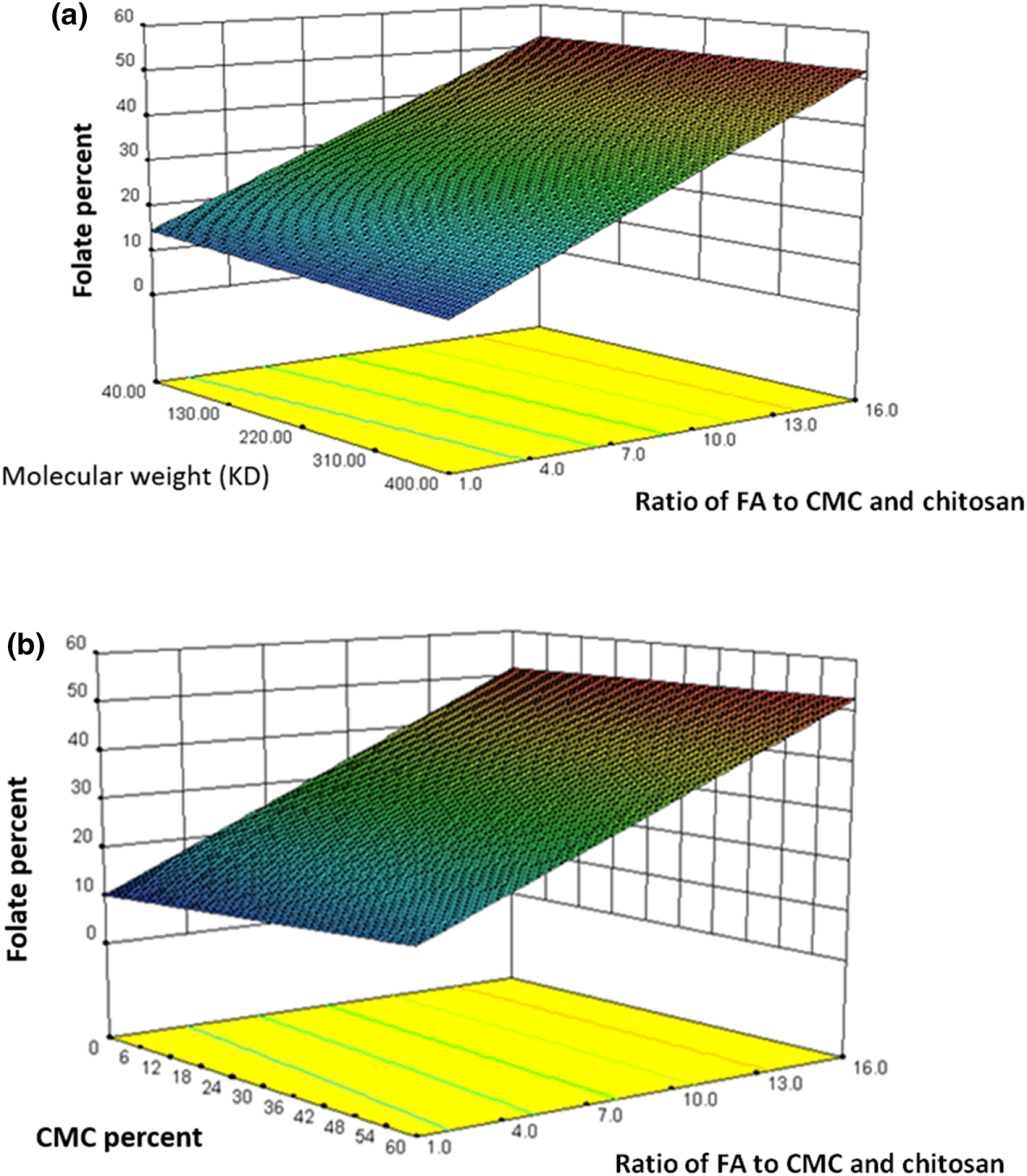
Response surface of percent of folate substitution in terms of **a** molecular weight and the ratio of folic acid to CMCS and chitosan, **b** the substitution of CMC and the ratio of folic acid to chitosan CMCS

**Fig. 5:**
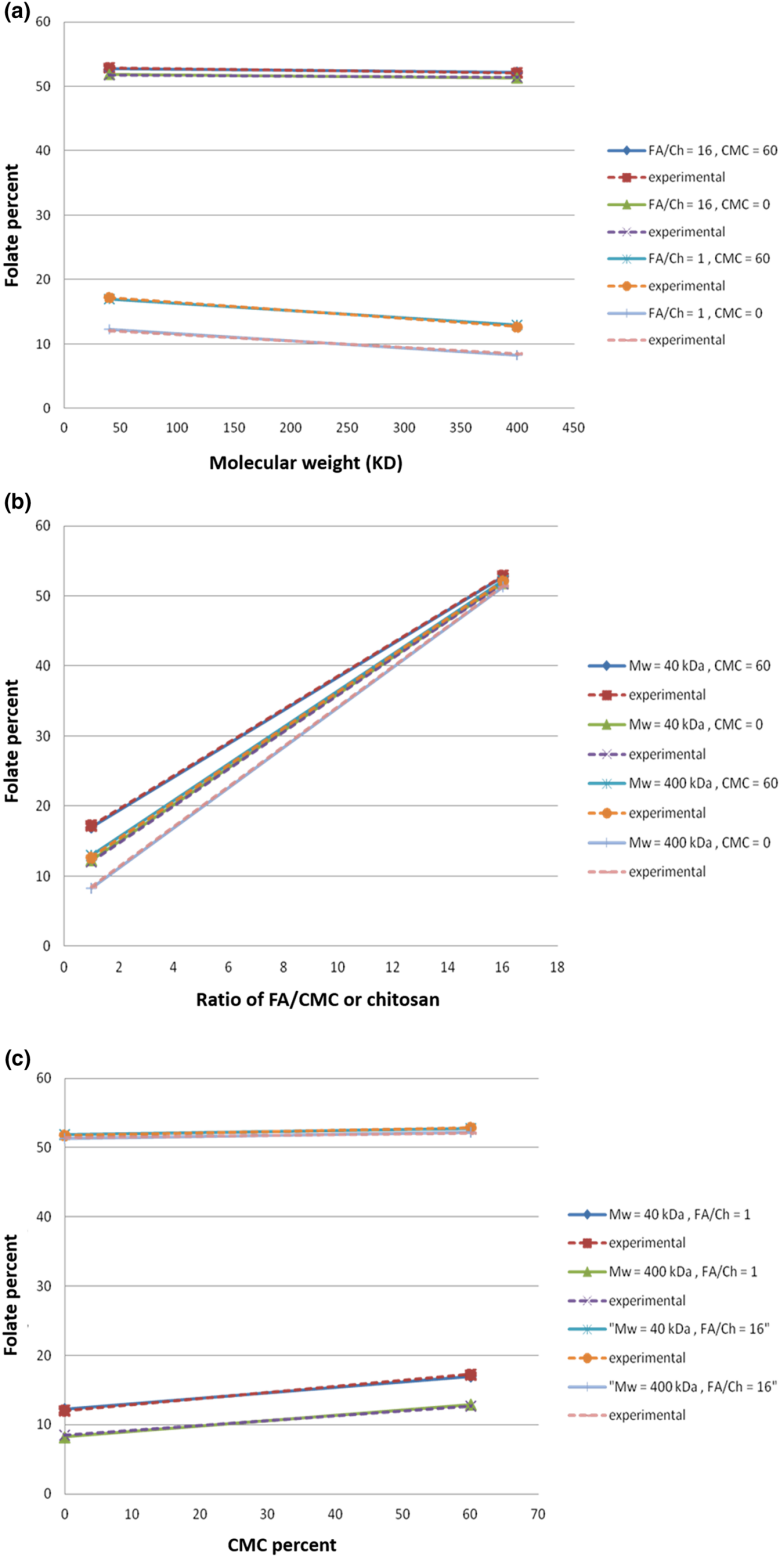
**a** Effect of molecular weight, **b** effect of different ratios of folic acid to CMCS and chitosan and **c** effect of CMC percentage on percent of folic acid substitution

Main effects and interactions of factors on the degree of substitution were observed by plotting its variations against the changes of each factor in two levels (low and high) of other factors using the data predicted by the generated model. Also, the experimental data for each factor are compared to the predicted plots (Figs. 4, 5).

By increasing the molecular weight, degree of FA substitution decreases and it is more significant in low molar ratios of folic acid (Fig. 5a).

As presented in Table 4, the molecular weight significantly affects the substitution of folate (*P* value less than 0.05) and it can be due to the increase in chain stiffness by increasing molecular weight and end to end distance of the chain (Möglich et al. 2006), resulting in less mobility of chitosan chains and leading to less availability of NH2 groups on chitosan chain to interact with folic acid.

Effect of different ratios of folic acid to chitosan and carboxymethyl chitosan on degree of FA substitution is depicted in Fig. 5b.

Based on ANOVA studies, ratios of folic acid to chitosan and carboxymethyl chitosan significantly affects the percentage of substitution of folic acid (*P* value less than 0.05) and its increments in both molecular weights result in higher degree of FA substitution. Clearly, higher folic acid concentration, higher availability of COOH groups of folic acid to NH_2_ groups of chitosan chains and thus the degree of FA substitution increases.

Figure 5c displays the effect of carboxymethyl groups on the degree of FA substitution. By introducing carboxymethyl groups to the chitosan chains, degree of FA substitution increases significantly (*P* value less than 0.05). This could be due to the presence of carboxylic acid groups and the phenomenon of proton motion. COOH groups of CMC can promote conversion of NH_2_ groups of chitosan to NH_3_^+^; therefore, their reactivity with COOH groups of folic acid increases (Larsson et al. 2013; Li et al. 2008).

## Conclusion

In this research, effect of molecular weight, degree of substitution of hydrophilic carboxymethyl groups and the molar ratio of folic acid to chitosan/carboxymethyl chitosan (CMCS), on the degree of FA substitution grafted on chitosan and CMCS chains using two-level factorial model was investigated. *P* value of proposed model was less than 0.05 and it shows that the proposed model is significant. The results showed that the molar ratio of folic acid to chitosan/CMCS has the highest positive impact on the degree of FA substitution. The comparison of experimental results and estimated data using the proposed model showed that this model has a good ability to estimate and optimize the FA substitution for chitosan and CMCS.
